# Binding Interaction of Betulinic Acid to α-Glucosidase and Its Alleviation on Postprandial Hyperglycemia

**DOI:** 10.3390/molecules27082517

**Published:** 2022-04-13

**Authors:** Shaodan Chen, Bing Lin, Jiangyong Gu, Tianqiao Yong, Xiong Gao, Yizhen Xie, Chun Xiao, Janis Yaxian Zhan, Qingping Wu

**Affiliations:** 1 Guangdong Provincial Key Laboratory of Microbial Safety and Health, Key Laboratory of Agricultural Microbiomics and Precision Application, Ministry of Agriculture and Rural Affairs, State Key Laboratory of Applied Microbiology Southern China, Institute of Microbiology, Guangdong Academy of Science, Guangzhou 510070, China; chensd@gdim.cn (S.C.); tianqiao@mail.ustc.edu.cn (T.Y.); gaoxiong@gdim.cn (X.G.); xieyz@gdim.cn (Y.X.); xiaochun960@hotmail.com (C.X.); 2 School of Pharmaceutical Science, Guangzhou University of Chinese Medicine, Guangzhou 510006, China; bing135388@163.com; 3 Research Centre for Integrative Medicine, School of Basic Medical Science, Guangzhou University of Chinese Medicine, Guangzhou 510006, China; gujy@gzucm.edu.cn

**Keywords:** betulinic acid, α-glucosidase, inhibition mechanism, postprandial hyperglycemia, synergistic effect

## Abstract

Inhibiting the intestinal α-glucosidase can effectively control postprandial hyperglycemia for type 2 diabetes mellitus (T2DM) treatment. In the present study, we reported the binding interaction of betulinic acid (BA), a pentacyclic triterpene widely distributed in nature, on α-glucosidase and its alleviation on postprandial hyperglycemia. BA was verified to exhibit a strong inhibitory effect against α-glucosidase with an IC_50_ value of 16.83 ± 1.16 μM. More importantly, it showed a synergistically inhibitory effect with acarbose. The underlying inhibitory mechanism was investigated by kinetics analysis, surface plasmon resonance (SPR) detection, molecular docking, molecular dynamics (MD) simulation and binding free energy calculation. BA showed a non-competitive inhibition on α-glucosidase. SPR revealed that it had a strong and fast affinity to α-glucosidase with an equilibrium dissociation constant (*K*_D_) value of 5.529 × 10^−5^ M and a slow dissociation. Molecular docking and MD simulation revealed that BA bound to the active site of α-glucosidase mainly due to the van der Waals force and hydrogen bond, and then changed the micro-environment and secondary structure of α-glucosidase. Free energy decomposition indicated amino acid residues such as PHE155, PHE175, HIE277, PHE298, GLU302, TRY311 and ASP347 of α-glucosidase at the binding pocket had strong interactions with BA, while LYS153, ARG210, ARG310, ARG354 and ARG437 showed a negative contribution to binding affinity between BA and α-glucosidase. Significantly, oral administration of BA alleviated the postprandial blood glucose fluctuations in mice. This work may provide new insights into the utilization of BA as a functional food and natural medicine for the control of postprandial hyperglycemia.

## 1. Introduction

Type 2 diabetes mellitus (T2DM) has become one of the major chronic diseases. According to International Diabetes Federation (IDF) 2019 estimation, around 463.0 million adults are suffering from T2DM, and this number will increase to about 578.4 million by 2030 and further around 700.2 million by 2045 [1]. Specially, T2DM is major featured with hyperglycemia, which leads to the occurrence of serious diabetes complications, such as vascular damage and cardiovascular disease [2,3]. Epidemiological studies suggested that postprandial hyperglycemia might be an independent risk factor of cardiovascular disease beyond and more powerful than fasting hyperglycemia for T2DM sufferers [4]. Thus, controlling postprandial hyperglycemia became an effective therapy in T2DM treatment and diabetes complications prevention.

Carbohydrate, such as starch and dextrin in foods, is the main source of human blood sugar. The α-glucosidase presented in the gastrointestinal tract can catalyze the cleavage of α-1,4 glycosidic bonds of carbohydrate to form glucose, which is absorbed by small intestine and then enters into blood circulation. Inhibiting the activity of intestinal α-glucosidase can effectively control postprandial hyperglycemia [5,6,7]. Acarbose, an α-glucosidase inhibitor, is effective in managing postprandial blood glucose; however, the side-effects have restricted its long-term applications [8]. Hence, searching for more effective and safer α-glucosidase inhibitors from natural sources is greatly demanded.

**Figure 1:**
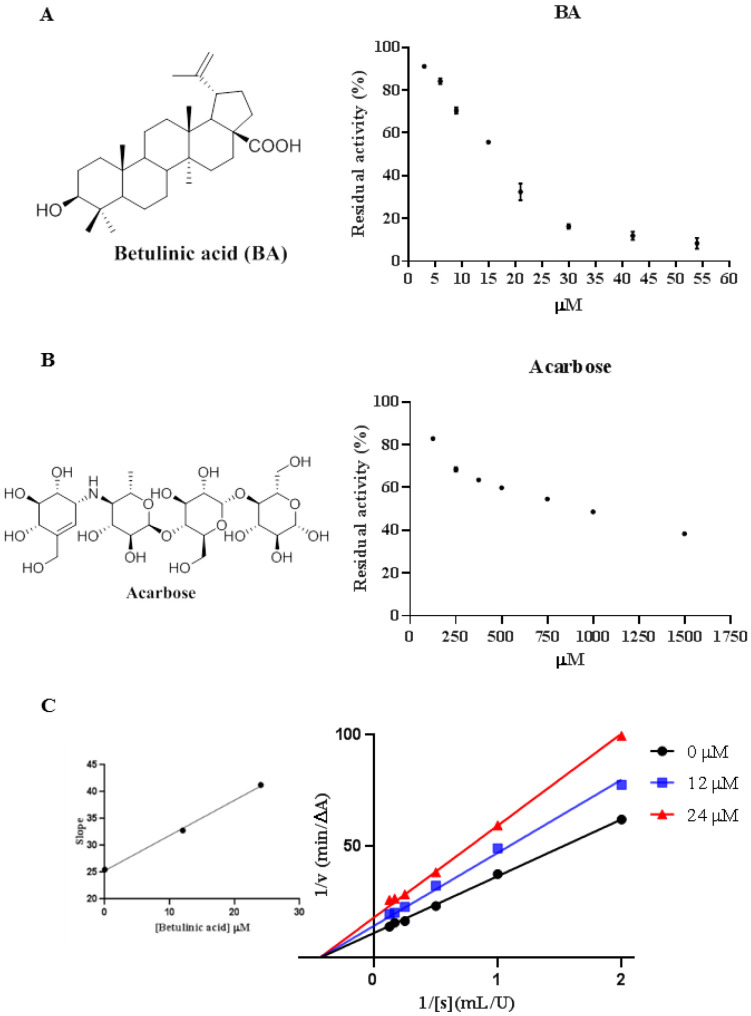
(**A**) Chemical structure of betulinic acid (**BA**) and its inhibitory effect of BA against α-glucosidase; (**B**) Chemical structure of acarbose and its inhibitory effect of acarbose against α-glucosidase; (**C**) Line-weaver-Burk diagrams (1/V vs. 1/[S]) for BA against α-glucosidase. The secondary plot of slope vs. BA was included.

Betulinic acid (BA, Figure 1A) is a triterpene widely presented in medicinal plants such as *Betula* (Betulaceae), *Ziziphus* (Rhamnaceae), *Syzygium* (Myrtaceae) and Chaga mushroom [9,10]. BA can be easily isolated from the source with different solvents or be synthesized by oxidation from betulin, a precursor with a yield of 20% dry weight in *Betula* species [9,11]. BA had a wide variety of bioactive effects including antitumor, anti-inflammatory, and hepatoprotective activities [12,13,14]. The regulation of BA in metabolic syndrome has gained more and more research in the past ten years and BA has been recorded as a potential antidiabetic agent [10,15,16,17,18,19,20,21,22]. Several studies have reported its regulatory effects on glucose absorption and uptake [23,24], insulin resistance [25], glucose production and glycogen synthesis [17,26,27,28]. BA was also found to inhibit α-glucosidase activity in vitro [7,29,30,31] and its inhibition mechanism was investigated by spectroscopic analysis and molecular docking [7]; however, the binding mechanism as well as the alleviated effect on postprandial hyperglycemia was still unclear.

In the present study, we firstly verified the inhibitory effect of BA and investigated its synergy with acarbose on α-glucosidase. The interaction mechanism was further explained by SPR analysis and molecular dynamics (MD) simulation in addition to molecular docking. Furthermore, the modulating effect of oral administration of BA on the postprandial blood glucose level was detected in vivo. This work provides novel insights into the use of BA as a functional food or a natural medicine for the control of hyperglycemia.

## 2. Results and Discussion

### 2.1. Inhibitory Effect of BA against α-Glucosidase

As shown in Figure 1A,B, BA and acarbose inhibited α-glucosidase activity in a dose-dependent manner. Compared to acarbose, BA displayed superior inhibition against α-glucosidase in very low concentrations, with IC_50_ value of 16.83 ± 1.16 μM, which was approximately 50-fold stronger than acarbose (841.3 ± 29.6 μM). Previous research had reported BA inhibited α-glucosidase in vitro with IC_50_ value from 10.6 to 83.6 μM, which was in accordance with our result [7,29,30]. Combined with previous papers [7,29,30,32,33,34], it was found that the different substitutions at C-3 and C-28 positions of BA could have influence on the inhibition activity. Additional polar interactions formed at C-3 and C-28 of BA would improve the inhibitory potency. 

### 2.2. Inhibition of Acarbose Combined with BA against α-Glucosidase

Acarbose, a drug based on carbohydrate-related structures, is effective in contrasting postprandial hyperglycemia. However, it causes adverse gastrointestinal effects when administered in accompany with a high-carbohydrate diet [35]. It was found that the combination of two or more inhibitors could act synergistically, having an inhibitory effect against α-glucosidase; thus, the interaction of acarbose combined with BA on α-glucosidase inhibition was investigated. Based on the results summarized in Table 1, the combination of acarbose with BA showed a synergistic effect on inhibiting α-glucosidase activity as the values of *V_ab_* − *V_c_* were below −0.1 at different concentrations. Acarbose was a competitive inhibitor, while BA was a non-competitive one; the two inhibitors may bind to the different sites of the active pocket of α-glucosidase. The presence of BA may enhance the binding affinity between α-glucosidase and acarbose, and lead to a strong joint inhibition. The result was similar to the combined interaction of corosolic acid or oleanolic acid (another two triterpenoids) and acarbose on α-amylase, and the combination of apigenin and acarbose on α-glucosidase [36,37]. Taking advantage of this synergistic effect, it would be possible to reduce the side effects of acarbose at a lower dose by combining with BA. A reduction in side effects and toxicity is considered as one of the rationales of drugs combinations [38]. BA exerted global antidiabetic effects mediated by specific mechanisms of action with low toxicity in addition to inhibiting intestinal α-glucosidase [21], while acarbose mainly acted on the gastrointestinal tract [39]. Combining compounds with different signaling pathways and non-overlapping side effects, BA and acarbose may allow for a more significant total efficacy with possibly fewer side effects, which resembles the “cocktail therapy”. Moreover, the poor water solubility and low bioavailability of BA limits its clinical application [40]. It is possible that modification of BA by introduction of acarbose moiety to the parent molecule would increase the efficacy as well as the water solubility and bioavailability. It would be worthwhile to investigate the physiological mechanisms as well as bioavailability, pharmacokinetics, pharmacodynamics and toxicity of the combination of BA and acarbose in detail. 

### 2.3. Inhibition Types of BA on α-Glucosidase

The Lineweaver-Burk plot was employed to analyze the inhibition type of BA against α-glucosidase. As shown in Figure 1C, the horizontal axis intercept (−1/*K_m_*) remained constant while the vertical axis intercept (1/*V_max_*) ascended with the increasing concentration of BA, demonstrating that the inhibition type of BA was non-competitive, which was the same as another two triterpenes ursolic acid and oleanolic acid, but was different from acarbose [2]. However, Ding et al. had reported a different result on inhibition type of α-glucosidase by BA [7]. The difference may be attributed to the different source (i.e., yeast species) of α-glucosidase. Moreover, it had been reported that non-competitive inhibition is sometimes considered as a special case of mixed-type inhibition when the competitive constant and uncompetitive constant are exactly the same [41]. The Michaelis constant (*K_m_*) was 2.272 and *V_max_* was 0.0907 μM/min. Furthermore, the α-glucosidase inhibition kinetic constant (*K_i_*) was calculated as 38.5 μM according to the secondary plot.

### 2.4. Surface Plasmon Resonance (SPR) Analysis

**Figure 2:**
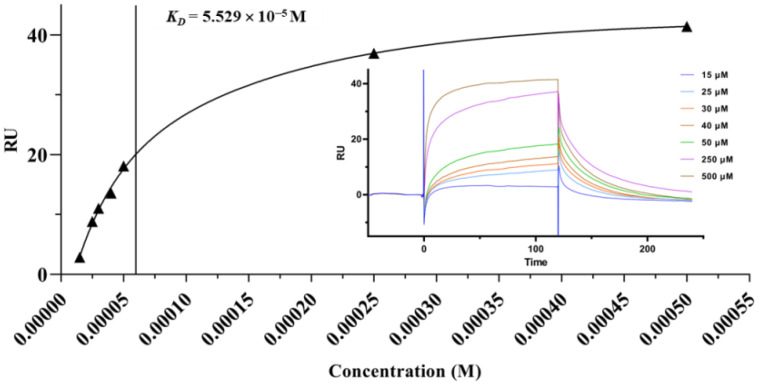
Binding kinetics and affinity of α-glucosidase to BA by SPR assay. Sensorgram showing the binding of BA at different concentrations to α-glucosidase was included.

SPR biosensor is a powerful tool to analyze the biomolecular binding interactions, as it can measure the kinetics and affinity of bimolecular binding in a real-time and label-free fashion with low reagent consumption [42]. As shown in Figure 2, BA effectively bound to α-glucosidase in a concentration-dependent manner. The binding time and the dissociation time were 120 s and 120 s, respectively. The equilibrium dissociation constant (*K*_D_) was 5.529 × 10^−5^ M, manifesting that BA had a high binding affinity to α-glucosidase and underwent a fast binding and slow dissociation reaction.

### 2.5. Molecular Docking

**Figure 3:**
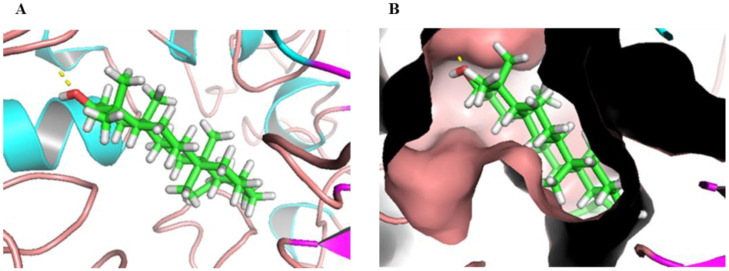
Molecular docking of BA with α-glucosidase. (**A**) Three-dimensional diagram of the interaction between BA and the binding pocket of α-glucosidase. (**B**) The receptor surface model of α-glucosidase with BA. BA was inserted into the hydrophobic cavity of α-glucosidase (light blue) in the surface structure; the yellow dashed line represents the hydrogen bond. The atoms of BA were color-coded as follows: C, green; H, white; O, red.

Molecular docking is a widely used structure-based drug design technique. It can predict the conformation of a ligand in the target binding site and calculate the binding energy [43]. The binding interaction of BA to α-glucosidase was analyzed through molecular docking. BA sat at the active pocket of α-glucosidase with high overlay, mainly due to van de Walls interaction (Figure 3). Moreover, BA formed an H-bond to Arg310 of α-glucosidase, which could further stabilize the orientation of the interaction. Thus, molecular docking demonstrated the binding interaction of BA to the hydrophobic cavity of α-glucosidase and the formation of hydrogen bonds together changed the micro-environment and structure of α-glucosidase, and led to a decrease in enzyme activity, which was in accordance with the results reported previously [7].

### 2.6. Molecular Dynamics (MD) Simulation and Calculation of Binding Free Energy

Molecular dynamics (MD) simulation is a prominent computational dynamics tool to analyze the molecular mechanism of an enzyme–ligand interaction. MD simulation can get the atomic trajectory at spatial and temporal scales, and provide detailed information about the conformational changes and fluctuations in protein [44,45].

**Figure 4:**
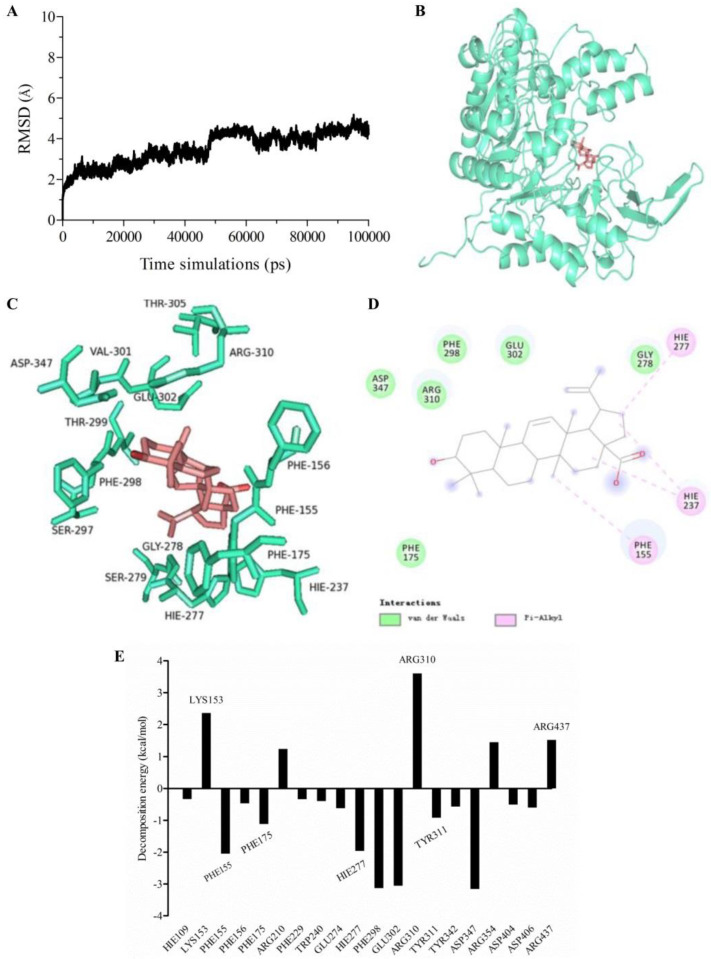
Molecular dynamics simulation. (**A**) Time dependence of root mean square deviation (RMSD) of the backbone of the protein in the complex; (**B**) Conformation of the binding of BA to α-glucosidase; (**C**) Interactions of BA to key residues of α-glucosidase; (**D**) 2D scheme of interactions of BA to key residues of α-glucosidase. Green and pink AA residues represent van der Waals interaction and Pi-alkyl interaction, respectively. (**E**) Decomposition of binding free energy of each residue for α-glucosidase.

The docked conformation based on molecular docking was used as initial conformation of MD simulation. The system was performed for 100 ns MD. The structural stability of enzyme-product complex was evaluated by calculation of the root mean square deviation (RMSD). RMSD measures the deviation of a set of coordinates of a protein to a reference set of coordinates. The RMSD for the protein backbones of α-glucosidase in complex with BA was shown in Figure 4A. Temporal RMSD was fluctuant initially and stable after 60 ns. RMSD for protein backbones converged to equilibrium during the last 5 ns. The average binding free energy for the system was calculated as −18.53 ± 3.44 kcal/mol based on the MD simulation by MM/GBSA method. For BA, the residues PHE175, ASP347, ARG310, PHE298, GLU302 and GLY278 presented van der Waals interactions to α-glucosidase. Meanwhile, HIE277, HIE237 and PHE155 contributed to Pi-alkyl interactions (Figure 4B–D). In order to further elucidate key amino acid residues, which had more contribution to the binding free energy, per-residue decomposition was used to generate the residue-product interaction spectra (Figure 4E). An amino acid residue may have a positive or negative contribution. The more negative the decomposed binding free energy of an amino acid residue means the more contributions to the binding affinity. Conversely, the more positive decomposed binding free energy of an amino acid residue, the stronger repulsive effect it will have. As shown in Figure 4E, conserved amino acid residues PHE155, PHE175, HIE277, PHE298, GLU302, TRY311 and ASP347 of α-glucosidase had strong interactions with the corresponding product in the complex, indicating that these amino acid residues would play important roles in the catalytic process. Consistent with the binding free energy analysis, most of the amino acid residues were hydrophobic, implying a hydrophobic–hydrophobic interaction. However, several amino acid residues, such as LYS153, ARG210, ARG310, ARG354 and ARG437, had repulsive effects on the binding of BA with α-glucosidase.

### 2.7. Effects of BA on Oral Saccharides Tolerance in Normal Mice

**Figure 5:**
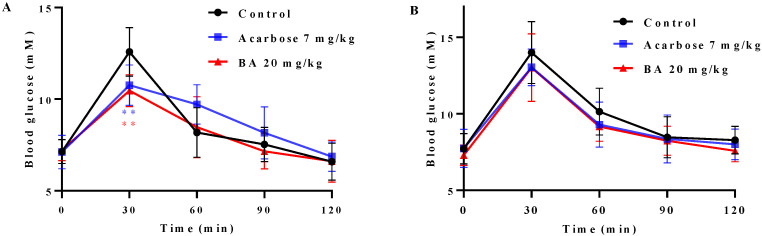
Effects of BA and acarbose on postprandial blood glucose levels in normal mice (*n* = 8). (**A**) Blood glucose after oral administration tests of mixed maltose and sucrose (each 1.0 g/kg); (**B**) Blood glucose after oral administration tests of glucose (2.0 g/kg). Data are expressed as means ± SD; ** *p* < 0.01 indicates a significant difference compared to control by *t*-test.

Postprandial hyperglycemia is the most important symptom of T2DM. BA had high binding affinity to α-glucosidase and effectively inhibited its activity, suggesting BA may influence the postprandial blood glucose level through inhibiting α-glucosidase activity. Accordingly, an oral disaccharide tolerance test (ODTT) and an oral glucose tolerance test (OGTT) were carried out in mice, respectively. As shown in Figure 5, the blood glucose level reached the highest point at 30 min after administration of maltose-sucrose or glucose and dropped sharply in the next 30 min. Compared to the control group, the postprandial blood glucose levels in BA-treated group and acarbose-treated group after oral administration of mixed maltose-sucrose were significantly suppressed (*p <* 0.01) (Figure 5A). In detail, the blood glucose levels at 30 min in BA-treated group and acarbose-treated group were decreased from 12.58 ± 0.47 to 10.46 ± 0.31 mM (*p <* 0.01) and to 10.76 ± 0.39 mM (*p <* 0.01), respectively, and then gradually decreased to the initial level. However, the BA-treated group (20 mg/kg) and acarbose-treated group (7 mg/kg) had no significant effect on blood glucose level in mice after oral glucose administration (Figure 5B). The results suggested that BA may slow down the hydrolysis rate of maltose and sucrose into glucose by inhibiting the activity of α-glucosidase, thereby delaying the absorption of intestinal glucose and effectively controlling the acute postprandial blood glucose fluctuation.

## 3. Materials and Methods

### 3.1. Materials

α-Glucosidase from yeast was purchased from Sigma-Aldrich (St. Louis, MO, USA). Acarbose (≥98%) and betulinic acid (≥98%) were obtained from Shanghai Yuanye Bio-Technology Co., Ltd. (Shanghai, China). *p*-Nitrophenyl-α-D-glucopyranoside (*p*NPG) were offered by Shanghai Aladdin Bio-Chem Technology Co., Ltd. (Shanghai, China). CM5 sensor chip, EDC/NHS and Amine Coupling Kit were purchased from GE Healthcare (Buckinghamshire, UK).

### 3.2. Inhibition Assay against α-Glucosidase

α-Glucosidase inhibition was assayed according to the methods described previously [46,47]. Briefly, 40 μL phosphate buffer (0.1 M, pH = 6.8), 10 μL BA solutions and 50 μL α-glucosidase solution (0.4 U/mL) were added into 96-well plates and mixed thoroughly. After being incubated at 37 °C for 10 min, 50 μL *p*NPG solution (2.5 mM) was added to initiate the reaction and stayed at 37 °C for 30 min. Then, 40 μL Na_2_CO_3_ solution (1.0 M) was added to terminate the reaction. A microplate reader was employed to read the absorbance at 405 nm. The inhibition rates (%) = [(OD_control_ − OD_control blank_) − (OD _test_ − OD_test blank_)]/(OD_control_ − OD_control blank_) × 100%.

### 3.3. Combination between BA and Acarbose

The interaction assay of acarbose with BA on α-glucosidase activity was performed as previously reported with minor modification [37,48]. The method was the same as the α-glucosidase activity assay. The OD value of the α-glucosidase reaction in the presence of acarbose divided by that obtained in its absence was named *V*_a_, which represents the remnant activity fraction of α-glucosidase after addition of acarbose. The value for BA (*V*_b_) was also defined. If the inhibitory effects on α-glucosidase of the two compounds are independent, the final remnant activity fraction of α-glucosidase (*V*_c_) is equal to *V*_a_*V*_b_ when the reaction is sequentially treated by those two compounds. The result of the OD value of the α-glucosidase reaction in the presence of both acarbose and BA divided by that obtained in their absence was named *V*_ab_. The types of interaction were determined by the relationship between *V*_ab_ and *V*_a_, *V*_b_, or *V*_c_. *V*_ab_ − *V*_c_ < −0.1 is considered as the synergistic (SY) interaction between BA and acarbose, −0.1 < *V*_ab_ − *V*_c_ < 0.1 means additive (AD) and *V*_ab_ − *V*_c_ > 0.1 is regarded as subadditive (SU). 

### 3.4. Enzyme Inhibition Kinetics

α-Glucosidase inhibition kinetics was studied as previously reported [47]. BA was set with the concentrations of 0, 12 and 24 μM for 0.2 U/mL α-glucosidase. A series of concentrations of *p*NPG were used as substrates. The inhibition type was determined by using the Lineweaver–Burk plots and its secondary equations. The kinetic parameters (*V_max_* and *K_i_*) were evaluated by the nonlinear regression of the Michaelis–Menton equation with GraphPad Prism 7.0. (https://www.graphpad.com/support/prism-7-updates/, accessed on 12 May 2021).

### 3.5. SPR Assay

SPR analysis was applied on a Biacore T200 system (GE Healthcare, Uppsala, Sweden). α-Glucosidase solution (50 μg/mL) was prepared and stayed at room temperature for 30 min. Then, the activated α-glucosidase was immobilized on a CM5 sensor chip pre-activated by EDC/NHS with an amine coupling kit at a flow rate of 10 μL/min. BA solution of different concentrations containing 5% DMSO was serially injected over α-glucosidase coated surface. PBS was used as the mobile phase with a flow rate of 30 μL/min. According to the 1:1 Langmuir model, the equilibrium dissociation constant (*K*_D_) was calculated by the Biacore evaluation software. (https://www.cytivalifesciences.com/en/us/shop/protein-analysis/spr-label-free-analysis/software/biacore-insight-evaluation-software-p-23528, accessed on 26 October 2016).

### 3.6. Molecular Docking

CDOCKER was used to conduct the docking. Briefly, BA was minimized energetically with MMFF94 force field for 5000 iterations until the minimum RMS gradient was below 0.01. The structure of α-glucosidase was modeled as per our previous report [47]. Then, the molecular docking was performed in a graphical interface of CDOCKER in Discovery Studio. CDOCKER_Energy was used to evaluate the docking results.

### 3.7. Molecular Dynamic (MD) Simulation and Free Energy Calculation and Decomposition

MD simulation was performed with Amber 16. The structure of α-glucosidase docked with BA was utilized as the initial structure. Gaussian 16 was used to optimize the geometries and calculate the electrostatic potentials of compound at the DFT/6-31G* level. Antechamber module in Amber 16 was exploited to generate the topology files of the ligand with the charge distribution calculated at DFT/6-31G* level. The complex was added with the AMBER ff14SB force field and dissolved with TIP3P water at the size of 15Å. The system was neutralized with 0.15 M NaCl. Then, the system was minimized by the sequence of solvent molecules, side chains of proteins and then backbone. The temperature was then increased gradually to 300 K. Dynamics simulation production was performed for 100 ns and the last 5 ns were extracted for analysis. The Molecular Mechanics/Generalized Born Surface Area (MM/GBSA) method was employed to calculate the binding free energy. The MM/GBSA method includes the calculation of the van der Waals interaction energy, the electrostatic energy, the non-polar solvation free energy and the polar solvation. The contribution of binding energy of each residue was further decomposed into four parts: van der Waals energy, electrostatic energy, non-polar solvation energy and polar solvation energy. The key residues which are responsible for the binding affinity of the complex can be discovered by this method [43,45].

### 3.8. Oral Disaccharide Tolerance Test (ODTT) and Oral Glucose Tolerance Test (OGTT)

The dynamic characteristics of postprandial blood glucose level were evaluated by ODTT and OGTT, which were performed as described previously [49]. BALB/c mice (5 weeks old, male) were purchased from Guangdong Medical Laboratory Animal Center (Guangzhou, China). The animal study was approved by the Committee on the Ethics of Animal Experiments of the Institute of Microbiology, Guangdong Academy of Science (GT-IACUC202012102). After one week of acclimation, the mice were randomly divided into a control group, BA-treated group and acarbose-treated group (*n* = 8), respectively. All the mice were fasted for 6 h, and then, the BA-treated and acarbose-treated groups were orally administered with BA (20 mg/kg) and acarbose (7 mg/kg), respectively, while the control group was given the same volume of distilled water. After 30 min, all the mice were intragastrically administered with the mixed maltose-sucrose solution (2 g/kg). In total, 10 μL of blood was collected by tail cut and glucose levels at 0, 30, 60, 90 and 120 min was measured by glucometer (Roche ACCU-CHEK Performa Nano), respectively. Two days later, all the mice were give the oral glucose tolerance test (OGTT), which was performed as the ODTT modus. The oral dose of glucose was also 2 g/kg.

### 3.9. Statistical Analysis

Data were performed by GraphPad Prism 7.0 software and the results were expressed as means ± SD. Comparison between groups was analyzed by Student’s *t*-test. *p* < 0.05 was considered to be significant.

## 4. Conclusions

In summary, the strong inhibitory effect of BA against α-glucosidase with an IC_50_ value of 16.83 ± 1.16 μM was verified and its non-competitive inhibition was investigated. Moreover, BA exerted a synergistically inhibitory effect combined with acarbose, which may allow reducing the side-effects of acarbose in T2DM treatment. More importantly, the underlying inhibitory mechanism of BA against α-glucosidase was explored by SPR analysis, molecular docking, molecular dynamics (MD) simulation and binding free energy calculation. SPR revealed that it had a strong and fast affinity to α-glucosidase with equilibrium dissociation constant (*K*_D_) value of 5.529 × 10^−^^5^ M and a slow dissociation. Molecular docking revealed that BA bound to the active site of α-glucosidase mainly due to the van der Waals force and hydrogen bond, and then changed the micro-environment and secondary structure of α-glucosidase. MD simulation and MM/GBSA binding free energy calculations further strengthened the evidence. Free energy decomposition indicated conserved amino acid residues such as PHE155, PHE175, HIE277, PHE298, GLU302, TRY311 and ASP347 had strong interactions with BA and α-glucosidase, which were crucial for the interactions of BA and α-glucosidase. Chemical modification of key amino acids would be conducted to verify the credibility of the molecular docking and MD modeling results. Additionally, oral administration of BA significantly suppresses postprandial blood glucose levels in normal mice. These findings may provide implications to understand the inhibitory mechanism of BA on α-glucosidase, and contribute to the development of natural effective inhibitors for diabetes management in the future. Future work will focus on the role of BA in intestinal glucose absorption and glucose homeostasis.

## Figures and Tables

**Table 1:** Synergistic effect of acarbose with BA on the inhibitory activity of α-glucosidase*.

	Acarbose-600 μM			Acarbose-800 μM			Acarbose-1000 μM		
	Value		Interaction	Value		Interaction	Value		Interaction
	*V_ab_*	*V_c_*	*V_ab_* − *V_c_*	*V_ab_*	*V_c_*	*V_ab_* − *V_c_*	*V_ab_*	*V_c_*	*V_ab_* − *V_c_*
BA-5 μM	0.47	0.59	−0.12SY	0.42	0.56	−0.15SY	0.41	0.53	−0.13SY
BA-10 μM	0.39	0.57	−0.18SY	0.36	0.54	−0.18SY	0.34	0.51	−0.17SY
BA-15 μM	0.32	0.50	−0.18SY	0.29	0.48	−0.18SY	0.28	0.45	−0.16SY

*V_ab_* and *V_c_* are, respectively, defined as the observed and expected residual activity treated by an acarbose–BA mixture. SY means synergistic interaction.

## Data Availability

The data are available from the corresponding author on reasonable request.

## Sample Availability

Not available.

## References

[1] Khursheed R., Singh S.K., Wadhwa S., Gulati M., Awasthi A. (2020). Therapeutic potential of mushrooms in diabetes mellitus: Role of polysaccharides. Int. J. Biol. Macromol..

[2] Ding H., Hu X., Xu X., Zhang G., Gong D. (2018). Inhibitory mechanism of two allosteric inhibitors, oleanolic acid and ursolic acid on α-glucosidase. Int. J. Biol. Macromol..

[3] Kato A., Hayashi E., Miyauchi S., Adachi I., Imahori T., Natori Y., Yoshimura Y., Nash R.J., Shimaoka H., Nakagome I. (2012). α-1-*C*-butyl-1,4-dideoxy-1,4-imino-ι-arabinitol as a second-generation iminosugar-based oral α-glucosidase inhibitor for improving postprandial hyperglycemia. J. Med. Chem..

[4] Esposito K., Giugliano D., Nappo F., Marfella R., Campanian Postprandial Hyperglycemia Study Group (2004). Regression of carotid atherosclerosis by control of postprandial hyperglycemia in type 2 diabetes mellitus. Circulation.

[5] Guo L., Yang C., Yang R., Zhao W. (2022). Magnetically anchored antibody-coupled nanocomposite as α-Amylase inhibitor for long-time protection against glycemic variability. Chem. Eng. J..

[6] Ma Y.Y., Zhao D.G., Zhang R.Q., He X., Li B.Q., Zhang X.Z., Wang Z.J., Zhang K. (2020). Identification of bioactive compounds that contribute to the α-glucosidase inhibitory activity of rosemary. Food Funct..

[7] Ding H., Wu X., Pan J., Hu X., Gong D., Zhang G. (2018). New insights into the inhibition mechanism of betulinic acid on α-glucosidase. J. Agric. Food Chem..

[8] Shah M.A., Khalil R., Ul-Haq Z., Panichayupakaranant P. (2017). α-Glucosidase inhibitory effect of rhinacanthins-rich extract from *Rhinacanthus nasutus* leaf and synergistic effect in combination with acarbose. J. Funct. Foods.

[9] Ali-Seyed M., Jantan I., Vijayaraghavan K., Bukhari S.N. (2016). Betulinic Acid: Recent Advances in Chemical Modifications, Effective Delivery, and Molecular Mechanisms of a Promising Anticancer Therapy. Chem. Biol. Drug Des..

[10] Luis Rios J., Manez S. (2018). New pharmacological opportunities for betulinic acid. Planta Med..

[11] Melnikova N., Burlova I., Kiseleva T., Klabukova I., Gulenova M., Kislitsin C.A., Vasin V., Tanaseichuk B. (2012). A practical synthesis of betulonic acid using selective oxidation of betulin on aluminium solid support. Molecules.

[12] Huang L., Zhu L., Ou Z., Ma C., Kong L., Huang Y., Chen Y., Zhao H., Wen L., Wu J. (2021). Betulinic acid protects against renal damage by attenuation of oxidative stress and inflammation via Nrf2 signaling pathway in T-2 toxin-induced mice. Int. Immunopharmacol..

[13] Harwansh R.K., Mukherjee P.K., Biswas S. (2017). Nanoemulsion as a novel carrier system for improvement of betulinic acid oral bioavailability and hepatoprotective activity. J. Mol. Liq..

[14] Serain A.F., Morosi L., Ceruti T., Matteo C., Meroni M., Minatel E., Zucchetti M., Salvador M.J. (2021). Betulinic acid and its spray dried microparticle formulation: In vitro PDT effect against ovarian carcinoma cell line and in vivo plasma and tumor disposition. J. Photochem. Photobiol. B-Biol..

[15] Zeng A., Hua H., Liu L., Zhao J. (2019). Betulinic acid induces apoptosis and inhibits metastasis of human colorectal cancer cells in vitro and in vivo. Bioorg. Med. Chem..

[16] Liao L., Liu C., Xie X., Zhou J. (2020). Betulinic acid induces apoptosis and impairs migration and invasion in a mouse model of ovarian cancer. J. Food Biochem..

[17] Kim S.J., Quan H.Y., Jeong K.J., Kim D.Y., Kim G.W., Jo H.K., Chung S.H. (2014). Beneficial effect of betulinic acid on hyperglycemia via suppression of hepatic glucose production. J. Agric. Food Chem..

[18] Ou Z., Zhao J., Zhu L., Huang L., Ma Y., Ma C., Luo C., Zhu Z., Yuan Z., Wu J. (2019). Anti-inflammatory effect and potential mechanism of betulinic acid on lambda-carrageenan-induced paw edema in mice. Biomed. Pharmacother..

[19] Gautam R., Jachak S.M. (2009). Recent developments in anti-inflammatory natural products. Med. Res. Rev..

[20] Kim J., Lee Y.S., Kim C.S., Kim J.S. (2012). Betulinic acid has an inhibitory effect on pancreatic lipase and induces adipocyte lipolysis. Phytother. Res..

[21] Silva F.S.G., Oliveira P.J., Duarte M.F. (2016). Oleanolic, ursolic, and betulinic acids as food supplements or pharmaceutical agents for type 2 diabetes: Promise or illusion?. J. Agric. Food Chem..

[22] Vinayagam R., Xiao J., Xu B. (2017). An insight into anti-diabetic properties of dietary phytochemicals. Phytochem. Rev..

[23] Kumar S., Kumar V., Prakash O. (2013). Enzymes inhibition and antidiabetic effect of isolated constituents from *Dillenia indica*. Biomed Res. Int..

[24] de Melo C.L., Queiroz M.G.R., Arruda Filho A.C.V., Rodrigues A.M., de Sousa D.F., Almeida J.G.L., Pessoa O.D.L., Silveira E.R., Menezes D.B., Melo T.S. (2009). Betulinic acid, a natural pentacyclic triterpenoid, prevents abdominal fat accumulation in mice fed a high-fat diet. J. Agric. Food Chem..

[25] Gomes Castro A.J., Silva Frederico M.J., Cazarolli L.H., Bretanha L.C., Tavares L.d.C., Buss Z.d.S., Dutra M.F., Pacheco de Souza A.Z., Pizzolatti M.G., Mena Barreto Silva F.R. (2014). Betulinic acid and 1,25(OH)_2_ vitamin D_3_ share intracellular signal transduction in glucose homeostasis in soleus muscle. Int. J. Biochem. Cell Biol..

[26] Wen X., Sun H., Liu J., Cheng K., Zhang P., Zhang L., Hao J., Zhang L., Ni P., Zographos S.E. (2008). Naturally occurring pentacyclic triterpenes as inhibitors of glycogen phosphorylase: Synthesis, structure-activity relationships, and X-ray crystallographic studies. J. Med. Chem..

[27] Ha D.T., Dao Trong T., Nguyen Bich T., Nhiem N.X., Ngoc T.M., Yim N., Bae K. (2009). Palbinone and triterpenes from *Moutan Cortex* (*Paeonia suffruticosa*, Paeoniaceae) stimulate glucose uptake and glycogen synthesis via activation of AMPK in insulin-resistant human HepG2 Cells. Bioorg. Med. Chem. Lett..

[28] Heiss E.H., Kramer M.P., Atanasov A.G., Beres H., Schachner D., Dirsch V.M. (2014). Glycolytic switch in response to betulinic acid in non-cancer cells. PLoS ONE.

[29] Choi C.I., Lee S.R., Kim K.H. (2015). Antioxidant and α-glucosidase inhibitory activities of constituents from *Euonymus alatus* twigs. Ind. Crops Prod..

[30] Tri M.D., Phat N.T., Trung N.T., Phan C.T.D., Minh P.N., Chi M.T., Nguyen T.P., Dang C.H., Hong Truong L., Pham N.K.T. (2021). A new 26-norlanostane from *Phlogacanthus turgidus* growing in Vietnam. J. Asian Nat. Prod. Res..

[31] Thengyai S., Thiantongin P., Sontimuang C., Ovatlarnporn C., Puttarak P. (2020). α-Glucosidase and α-amylase inhibitory activities of medicinal plants in Thai antidiabetic recipes and bioactive compounds from *Vitex glabrata* R. Br. stem bark. J. Herb. Med..

[32] Chukwujekwu J.C., Rengasamy K.R., de Kock C.A., Smith P.J., Slavetinska L.P., van Staden J. (2016). α-glucosidase inhibitory and antiplasmodial properties of terpenoids from the leaves of *Buddleja saligna* Willd. J. Enzym. Inhib. Med. Chem..

[33] Kazakova O.B., Giniyatullina G.V., Mustafin A.G., Babkov D.A., Sokolova E.V., Spasov A.A. (2020). Evaluation of Cytotoxicity and α-Glucosidase Inhibitory Activity of Amide and Polyamino-Derivatives of Lupane Triterpenoids. Molecules.

[34] Khusnutdinova E.F., Petrova A.V., Thu H.N.T., Tu A.L.T., Thanh T.N., Thi C.B., Babkov D.A., Kazakova O.B. (2019). Structural modifications of 2,3-indolobetulinic acid: Design and synthesis of highly potent α-glucosidase inhibitors. Bioorg. Chem..

[35] Cardullo N., Floresta G., Rescifina A., Muccilli V., Tringali C. (2021). Synthesis and in vitro evaluation of chlorogenic acid amides as potential hypoglycemic agents and their synergistic effect with acarbose. Bioorg. Chem..

[36] Zhang B., Xing Y., Wen C., Yu X., Sun W., Xiu Z., Dong Y. (2017). Pentacyclic triterpenes as α-glucosidase and α-amylase inhibitors: Structure-activity relationships and the synergism with acarbose. Bioorg. Med. Chem. Lett..

[37] Yang J., Wang X., Zhang C., Ma L., Wei T., Zhao Y., Peng X. (2021). Comparative study of inhibition mechanisms of structurally different flavonoid compounds on α-glucosidase and synergistic effect with acarbose. Food Chem..

[38] Mabate B., Daub C.D., Malgas S., Edkins A.L., Pletschke B.I. (2021). A Combination Approach in Inhibiting Type 2 Diabetes-Related Enzymes Using Ecklonia radiata Fucoidan and Acarbose. Pharmaceutics.

[39] Martin A., Montgomery P. (1996). Acarbose: An α-glucosidase inhibitor. Am. J. Health-Syst. Pharm..

[40] Oboh M., Govender L., Siwela M., Mkhwanazi B.N. (2021). Anti-Diabetic Potential of Plant-Based Pentacyclic Triterpene Derivatives: Progress Made to Improve Efficacy and Bioavailability. Molecules.

[41] Kan L., Capuano E., Fogliano V., Verkerk R., Mes J.J., Tomassen M.M.M., Oliviero T. (2021). Inhibition of α-glucosidases by tea polyphenols in rat intestinal extract and Caco-2 cells grown on Transwell. Food Chem..

[42] Zhou J., Qi Q., Wang C., Qian Y., Liu G., Wang Y., Fu L. (2019). Surface plasmon resonance (SPR) biosensors for food allergen detection in food matrices. Biosens. Bioelectron..

[43] Wu Z., Xu H., Wang M., Zhan R., Chen W., Zhang R., Kuang Z., Zhang F., Wang K., Gu J. (2019). Molecular docking and molecular dynamics studies on selective synthesis of α-Amyrin and β-Amyrin by oxidosqualene cyclases from *Ilex asprella*. Int. J. Mol. Sci..

[44] Zhang L., Wang P., Yang Z., Du F., Li Z., Wu C., Fang A., Xu X., Zhou G. (2020). Molecular dynamics simulation exploration of the interaction between curcumin and myosin combined with the results of spectroscopy techniques. Food Hydrocoll..

[45] Ni M., Hu X., Gong D., Zhang G. (2020). Inhibitory mechanism of vitexin on α-glucosidase and its synergy with acarbose. Food Hydrocoll..

[46] Chen S.D., Yong T.Q., Xiao C., Su J.Y., Zhang Y.F., Jiao C.W., Xie Y.Z. (2018). Pyrrole alkaloids and ergosterols from *Grifola frondosa* exert anti-α-glucosidase and anti-proliferative activities. J. Funct. Foods.

[47] Chen S.D., Yong T.Q., Xiao C., Gao X., Xie Y.Z., Hu H.P., Li X.M., Chen D.L., Pan H.H., Wu Q.P. (2021). Inhibitory effect of triterpenoids from the mushroom *Inonotus obliquus* against α-glucosidase and their interaction: Inhibition kinetics and molecular stimulations. Bioorg. Chem..

[48] Cai S., Wang O., Wang M., He J., Wang Y., Zhang D., Zhou F., Ji B. (2012). In vitro inhibitory effect on pancreatic lipase activity of subfractions from ethanol extracts of fermented Oats (*Avena sativa* L.) and synergistic effect of three phenolic acids. J. Agric. Food Chem..

[49] Zhu B., Li M.Y., Lin Q., Liang Z., Xin Q., Wang M., He Z., Wang X., Wu X., Chen G.G. (2020). Lipid oversupply induces CD36 sarcolemmal translocation via dual modulation of PKC zeta and TB1CD1: An early event prior to insulin resistance. Theranostics.

